# Supplementary material for: Family Needs Checklist: Development of a Mobile Application for Parents with Children to Assess the Risk for Child Maltreatment
Source: Int J Environ Res Public Health. 2022 Aug 9;19(16):9810. doi: 10.3390/ijerph19169810 (PMC9408053; doi:10.3390/ijerph19169810)
Supplement: Supplementary file 1 [file ijerph-19-09810-s001.zip › Supplementary mateial_ Table S4_ Rantanen et al 2022 manuscript.pdf]

Supplementary material

Table S4: Initial Family Needs Checklist (FNC) online application, feedback summary, and modifications to the final version.

section 1 Demographic information (removed as such. Items included in other sections of the FNC)			
Statement	Contents/answer	Feedback summary	Modifications to the final version
Your age	≤18		Statement no. 5 in the final version.
Gender	male<br>female	Importance of gender?	removed<br>Instead of highlighting the gender, all parents or caregivers are included in the feedback section.
Education	- No formal (or low) education<br>- Vocational degree<br>- College degree<br>- University degree		Statement no. 6 in the final version
Family status	-married, civil partnership<br>-Single<br>-Divorced<br>- Reconstituted family<br>- Widow	-In some areas there are a lot of same-gender couples – should that be reflected?<br>-There are also civil partnerships<br>- The question of family status is, in fact, a question of marital status and does not in itself refer to family status. Divorced parents can have effective joint custody, parents can live in cohabitation, and so on<br>- The question on family status has widow as an option, not widower would suggest it is aimed at women	Statement no.16 in the final version. Risk factors, namely single parenthood, divorced family and lack of another biological parent are included in the statement and/or feedback
Number of children	≥3		→Statement No. 17 in the final version
Age of all the children in years (for example 0,1,3)	≤3		→ statement no.19 in the final version
Twins in the family<br>Premature baby<br>Gender of children	Yes-No<br>Yes-No<br>Girl-Boy	Also, any risk factors linked to a child's gender – should be adjusted culturally?	Risk factors for Shaken baby syndrome have been moved to the info section

		The gender of the child may also be other than girl or boy	
Section 2 concerning the Child risk factors<br>Think about the situation with your child (Section 2 is moved to section 5 in the final checklist → Giving priority to parents and the family situation)			
My child had complications associated with pregnancy or birth	Yes-No	Prematurity can also be caused by abuse and vice versa	→ statement no. 23 in the final version. Feedback is considered in the feedback section.
My child has been diagnosed with a developmental or physical illness or has challenges related to emotions or social situations	Yes-No	What about a health condition?	→ statement no. 25 in the final version. Poor health is considered in the feedback of statements no. 13, 14 and 15
I know what shaking can cause to the baby	Yes-No	-We're not sure about including this as the app is screening for current/potential issues – health promotion would be part of a different checklist where all areas would be covered in a discussion with a practitioner i.e. SIDS/managing crying/substance use/home safety etc.	→ FNC design change. We added an information section because FNC is an anonymous self-referral instrument. This subject has been moved to the info section in the final FNC version.
My child is weeping a lot	Yes-No	Cries a lot?	→ Wording changed → statement no. 24 in the final version
My child is often disobedient, misbehaving, difficult or irritable	Yes-No	-Consider taking out the terms disobedient and misbehaving.<br>-Differences in temperament between the child and the parent can be a risk factor. Especially when the child is calm and slow-paced and the parent vice versa	→ statement no 26 in the final version. All words are included for multicultural purposes.<br>Feedback is considered in the feedback section.
My child has challenges at school or daycare	Yes-No	Also, my child has challenges at school, most children do face challenges at school I can say yes to that for all 3 of my children	→ statement no 27 in the final version. Feedback is considered in the feedback section.
My child is often sick or unwell	Yes-No	-could experience this matter differently. The experience of such frequent - words raises in parents the question of what is often	→ amalgamed to statement no.12 in the final version. A child with chronic illness is also considered in the feedback section. The word "often" is left in place because it has

			a special meaning. The purpose is clarified in the feedback section.
I have to calm my child by giving drugs, sedatives or other substances	Yes-No	-controversial/leading?<br>-concerned about the wording of prescribed drugs?<br>-Substances→ alcohol?<br>-I have to calm my child with drugs - can the abuse described in the question even invite abuse?	→ statement no 28 in the final version<br>All words are included for multicultural purposes. Feedback is considered in the feedback section.
Section 3 concerning the parent or caregiver risk factors<br>Think about the situation with yourself (Section is moved to the top of the statement sections)			
Although I became pregnant, I feel I did not want to	Yes-No		→ Amalgamed into the subjects of parent's young age, substance abuse, mental health problems and financial problems
I have a criminal record	Yes-No	A strong statement, can we ask this on a low threshold?	→ Wording changed → statement no 10 in the final version. Feedback is considered as the FNC is an anonymous self- referral instrument.
I feel lonely and haven't had enough support from the community, relatives, friends or spouse	Yes-No	Lack of support is considered an important risk factor	→ statement no. 21 in the final version
I have missed my child's health appointments	Yes-No	The reason for not using appointments times is important to discuss	→ Removed and included in the info section "neglect"
I use child and family services regularly as recommended	Yes-No	This is not a risk factor	→ This is reversed mode of the statement" I have missed my child's health appointments". Removed
I have to use a lot of health services with my child	Yes-No	Is this a risk factor?<br>Using a lot of health services is considered an important risk factor for FII	→added: "…because my child gets often sick or unwell", for a distinction between FII (Fabricated or Induced Illness) and "normal" frequent appointments. Statement no. 12 in the final version

I know what child maltreatment means	Yes-No	We're not sure about including this as the app is screening for current/potential issues – health promotion would be part of a different check-list where all areas would be covered in a discussion with a practitioner ie SIDS/managing crying/substance use/home safety etc	→ FNC design change. We added an information section because FNC is an anonymous self-referral instrument. This subject has been moved to the info section in the final FNC version
I know what child sexual violence means	Yes-No	We're not sure about including this as the app is screening for current/potential issues – health promotion would be part of a different check-list where all areas would be covered in a discussion with a practitioner ie SIDS/managing crying/substance use/home safety etc	→ FNC design change. We added an information section because FNC is an anonymous self-referral instrument. This subject has been moved to the info section in the final FNC version
I have been maltreated as a child	Yes-No	We're not sure ethically that you can ask a question like this without ensuring that there are proper support structures in place	→ FNC design change. FNC is an anonymous self-referral instrument. We included support structures in the FNC. Statement no. 1 in the final version
I have experienced traumatic events as a child and cannot get over them	Yes-No	And I don't want to get over them (in the Finnish language) .. a different thing to lay out the question so that I have a hard time getting over them	→ The wording has been improved. Statement no. 2 in the final version
I know what is the safe limit for alcohol use in a family with children	Yes-No	-Focus on parental public health knowledge. We're not sure about including this as the app is screening for current/potential issues – health promotion would be part of a different checklist where all areas would be covered in a discussion with a practitioner ie SIDS/managing crying/substance use/home safety etc.<br>- This is the only statement regarding drug issues and alcohol whether is sufficient. I am also thinking about the layout of the question, a safe limit for daily alcohol use…	→wording changed. FNC design change. FNC is an anonymous self-referral instrument. → statement no. 9 in the final version<br>NOTE: One more statement (no.8) was added regarding tobacco drugs or alcohol use
I am suffering from a serious illness	Yes-No	-I have a diagnosed physical illness or mental health issue? Amalgamation suggestion	→ Amalgamed to statement no. 11

		- should this be opened more; what is a serious illness	
I am suffering from mental health problems for example depression or a feeling of worthlessness	Yes-No	-I am suffering from a serious illness? Amalgamation suggestion<br>-Is there a difference between somatic and mental illness. Depression is also a disease and not a "mental health challenge". Could it be, for example, that I have health challenges that are stressful, maybe somatic or mental	→ statement no. 11 in the final version
I am living in stressful times	Yes-No		→ statement no. 7 in the final version
I often have to cancel my health or social appointments	Yes-No	Many of the questions need to be opened (reasoned), e.g. this statement	→ included in the feedback of the statement no. 1, 8, 9 and 21 in the final version
I know what child corporal punishment means	Yes-No	-we're not sure about including any of these as the app is screening for current/potential issues – health promotion would be part of a different checklist where all areas would be covered in a discussion with a practitioner ie SIDS/managing crying/substance use/home safety etc.<br>- Could there be an I accept corporal punishment (and an explanation of what corporal punishment means in the infobox)	→ This subject has been moved to the info section
I spend a lot of time on the internet or phone while taking care of my child	Yes-No	Is there another way of putting it?	→ This subject has been moved to the info section
I sometimes have inaccurate expectations about what the child's behavior should be like	Yes-No	What this question is getting at…child development?	→ statement no 3 in the final version. The purpose is clarified in the feedback section.
Parenting stresses me out	Yes-No	I find parenting difficult/exhausting???	→ removed, repetition of statement no 22
I have called my child names like stupid, ugly, fat or worthless	Yes-No	Controversial/leading and could be drawn out from the response to the previous question or the final question re verbally or physically lashing out wouldn't expect parents to admit to this	→ FNC design change. We added an information section because FNC is an anonymous self-referral instrument. This subject has been

			moved to the info section in the final FNC version
I have destroyed/harmed my child's favorite object for example toy or pet	Yes-No	Controversial/leading and could be drawn out from the response to the previous question or the final question re verbally or physically lashing out<br>add the word intentionally into the question	→ FNC design change. We added an information section because FNC is an anonymous self-referral instrument. This subject has been moved to the info section in the final FNC version
I cannot always control my child's disobedient behavior	Yes-No	-Controversial/leading and could be drawn out from the response to the previous question or the final question re verbally or physically lashing out<br>-What remains to be thought about here is what is meant by control? Own or use a child in a situation, or both	→wording changed→ statement no 4 in the final version. Feedback is considered in the feedback section.
I think that my child needs me all the time	Yes-No	clingy or lacks self-confidence?	→ removed →repetition (Fabricated or Induced Illness)
I was separated from my baby after the birth	Yes-No	This is in the wrong section. it is among maltreatment statements however often it is something that is completely out of the control of the parent and can be quite a traumatic experience to raise especially within such a section	→ Amalgamed with statement no. 23 in the final version
I have too little time to spend together with my child	Yes-No	Suggestion: I don't have enough time with my child?	→ amalgamed with statement no. 16 and 17 in the final version
I think that my child feels safe to talk openly about sensitive issues at home	Yes-No		→ This subject has been moved to the info section
I am able to share my love equally with my children	Yes-No	What does this refer to?	→ removed, repetition many children
I take care of my child's activities of daily living for example dental hygiene, general hygiene, healthy and nutritious nourishment or sleeping times	Yes-No	there's a lot here	→ wording changed and divided into possible neglect of health and physical care, social and emotional care and learning and supervision in statements no. 13,14 and 15 in the final version

I have threatened my child with abandonment or violence for example spanking or pulling hair	Yes-No	controversial and leading<br>perhaps one question such as: Sometimes I verbally or physically lash out at my child?	→ FNC design change. We added an information section because FNC is an anonymous self-referral instrument. This subject has been moved to the info section in the final FNC version
I tend to bring out my child's weaknesses in public or front of my child	Yes-No	controversial and leading<br>perhaps one question such as: Sometimes I verbally or physically lash out at my child?	→ FNC design change. We added an information section because FNC is an anonymous self-referral instrument. This subject has been moved to the info section in the final FNC version
I tend to punish my child verbally or physically	Yes-No	controversial and leading<br>perhaps one question such as: Sometimes I verbally or physically lash out at my child?	→ FNC design change. We added an information section because FNC is an anonymous self-referral instrument. This subject has been moved to the info section in the final FNC version
I tend to belittle my child	Yes-No	controversial and leading<br>perhaps one question such as: Sometimes I verbally or physically lash out at my child?<br>I wouldn't expect parents to admit to this	→ FNC design change. We added an information section because FNC is an anonymous self-referral instrument. This subject has been moved to the info section in the final FNC version
I punish my child for bad behavior or bad actions	Yes-No	controversial and leading<br>perhaps one question such as: Sometimes I verbally or physically lash out at my child?	→ FNC design change. We added an information section because FNC is an anonymous self-referral instrument. This subject has been moved to the info section in the final FNC version
I had to discipline my child by causing the child feel pain	Yes-No	controversial and leading<br>perhaps one question such as: Sometimes I verbally or physically lash out at my child?	→ FNC design change. We added an information section because FNC is an anonymous self-referral instrument. This subject has been

			moved to the info section in the final FNC version
We use traditional punishing methods at home, for example threatening, bribing, blackmailing or “naughty bench”	Yes-No	-controversial and leading<br>-perhaps one question such: Sometimes I verbally or physically lash out at my child?<br>- How is disciplinary education interpreted? Does everyone interpret this way? Any info on this?<br>-Naughty bench – is it the same as other examples? Is it child maltreatment?<br>-I wouldn't expect parents to admit to this<br>big difference between bribery and the naughty step	→ FNC design change. We added an information section because FNC is an anonymous self-referral instrument. This subject has been moved to the info section in the final FNC version
I think that there is a “normal” level of violence in parenting that is acceptable in our society	Yes-No	I believe it is appropriate in our society to use some form of disciplinary education	→ removed, parental violence information is given in the info section
Think about your family situation (section 4)			
One or more of my family members are unemployed	Yes-No		→ Amalgamed and included in a statement no. 18 in the final version
Our home is too cramped	Yes-No		→ Amalgamed and included in a statement no. 18 in the
We use a lot of welfare systems services for example benefits, public assistance or social work	Yes-No	-two questions?<br>Suggestion: We are in receipt of benefits/We have a social worker<br>- The wording is a bit negative<br>- Does our family use a lot of social services? What is a lot? What kind of? Have you applied for help yourself? Will this forcibly become a negative echo?	→ Amalgamed and included in a statement no. 18 in the final version. Wording changed.
We have a history of child protective interventions	Yes-No		→ Removed. There are currently conflicting results regarding the association between substantiation status and risk for future child maltreatment [117]
I think that my home is safe and healthy for a child to develop	Yes-No	focus on parental public health knowledge	→ FNC design change. We added an information section because FNC is

		we're not sure about including this as the app is screening for current/potential issues – health promotion would be part of a different check-list where all areas would be covered in a discussion with a practitioner ie SIDS/managing crying/substance use/home safety etc	an anonymous self-referral instrument. This subject has been moved to the info section in the final FNC version
I know what family violence means	Yes-No	we're not sure about including any of these as the app is screening for current/potential issues – health promotion would be part of a different check-list where all areas would be covered in a discussion with a practitioner ie SIDS/managing crying/substance use/home safety etc	→ FNC design change. We added an information section because FNC is an anonymous self-referral instrument. This subject has been moved to the info section in the final FNC version
My family has problems getting along	Yes-No		→ FNC design change. We added an information section because FNC is an anonymous self-referral instrument. This subject has been moved to the info section in the final FNC version
My child has been subjected to family violence	Yes-No		→ wording changed → statement no 22 in the final version
My partner sometimes says or does things that make me sad, depressed, stressed out or fearful	Yes-No	-could intimate partner violence statements be amalgamated into one question?<br>-if parents are filling in the questionnaire together, would the abused partner be willing to open up about it?<br>-what does sometimes mean?<br>-might drive abuse more underground, however, these questions might plant a seed of thought in the parents' minds and then later when they're ready they can access services<br>-It is difficult to draw a line between intimate partner violence and normal intimate partner arguments	→ Wording changed. Amalgamed into statement no. 21 in the final version. Feedback is considered in the feedback section.

		- Everyone's partners probably sometimes say things that make the other sad	
My partner sometimes keeps me from doing my favorite things like seeing friends or attending learning courses	Yes-No	-could intimate partner violence statements be amalgamated into one question?<br>-if parents are filling in the questionnaire together, would the abused partner be willing to open up about it?<br>-what does sometimes mean?<br>-might drive abuse more underground, however, these questions might plant a seed of thought in the parents' minds and then later when they're ready they can access services<br>-It is difficult to draw a line between intimate partner violence and normal intimate partner arguments	→ Wording changed and amalgamed into statement no. 21 in the final version
My partner has kept me from seeking medical or mental health care	Yes-No	-could intimate partner violence statements be amalgamated into one question?<br>-if parents are filling in the questionnaire together, would the abused partner be willing to open up about it?<br>-what does sometimes mean?<br>-might drive abuse more underground, however, these questions might plant a seed of thought in the parents' minds and then later when they're ready they can access services<br>-It is difficult to draw a line between intimate partner violence and normal intimate partner arguments	→ Wording changed and amalgamed into statement no. 21 in the final version

General feedback from the training participants

- The main point was the wording of the items (similar to feedback from the other countries). Most participants thought that the items were too "offensive/straightforward").
- We (one group) find the claims quite aggressive and guiding the parent
- In the title "family needs checklist" some of the questions are quite intrusive and I would see it more as a deep dive rather than a checklist.
- There perhaps should be some positive statements to respond to. Some of the statements seem negative
- I agree it may be useful to have in a toolkit for assessment but it would need some refining.
- Some of the statements may be difficult to understand?

- Participants were afraid that parents will not come back to the counseling situation or felt affronted.
- The order of the items should be modified, perhaps sorted by different headings
- Most participants preferred to use the checklist as a basis for a kind of interview, not as a self-completed questionnaire for parents
- The questions regarding knowledge should address not “child maltreatment” in a general term, but concrete behavior (e.g. slap in the face, smack on the bottom, call names to your child, embarrass your child in public,…)
- They thought that the checklist would be useful for professionals as a reminder and could be used for preparing a conversation with parents/caregivers.
- In comparison to tools being used in our country they overall sound very negative. Seem to blame parents. In our country there is more focus on strengths and resilience
- Parents need a certain level of emotional vulnerability to answer these questions, thus there is concern from trainees about parents during it on their own or receiving sufficient support afterward.
- Lack of clarity in what would happen if the parents answer “yes”. Could the children be taken away or have other serious consequences? This led to questioning the truthfulness of responses from the parents and openness about their situation. A key issue in this is if parents want help. Not clear how they would answer questions if they don’t want help or don’t want to admit issues in their family.
- Good questions are the ones that ask about parents’ experiences themselves.
- Even with the cover letter, it will be difficult to reassure the parent that this is a positive thing and for them to be honest
- We have found that children are very honest and reflective with a virtual wellbeing check-in. I'm sure this would be the case with this once the app is launched. It is a starting point for intervention with people that would not necessarily have been flagged up already.
- We felt as professionals we would need to have a very good relationship and trust with a family to be able to approach these questions with them
- Go in with a completely non-judgemental approach so the parent feels relaxed and will give more truthful answers
- The language used is not like any other language used in our country. It may be cultural or because it’s been translated but it seemed very abrupt.
- Do parents completing the checklist know that the purpose of it is to identify risks of child maltreatment? if so will they be truthful, if not is that ethical? Also had thoughts about what support there was after completion of the checklist as some of the questions might have left the parent feeling very vulnerable and low.
- The questions were good in reflecting the risk factors. Here again, came to mind, was one of the risk factors for the mother to have had previous uterine deaths or lost children? If I remember correctly, should it be in the claims?
- We discussed that the parent's own situation/experiences are directly related to the emotional abuse experienced by the child (“think of your own situation” in some of the statements). Caregivers unconsciously or consciously transfer their feelings/experiences to the child.
- Parents also need help - not just a child.
- We must understand the parent, even if we do not accept the parent's behavior.
- How can we support families more effectively?
- How could support for families in early childhood education be shown?
- Should there also be more protective factors?
- Should there be any feedback for even positive answers?

- Is the answer always honest or, despite a good answer, is there anything left to think about?
- How hard is it to go through 55 statements? Would you be able to split and get some feedback and later even finish?
- The cover letter is too long.
- The feedback related to young parental age is quite long but fortunately positive.
- Would it be good to proceed chronologically from the defendant's current condition and childhood to the affairs of your child and the affairs of the whole family? Subtitle for clarity in the statements.
- There is a lot to think about, and they are really good stimuli for discussion and are sure to provoke a lot of thoughts.
- Feedbacks are wonderfully safe and positive, encouraging and permissive, as it is certainly easier to receive feedback.
- It certainly helps to start a conversation, for example, between a parent and an employee, and also to take the conversation forward when the parent already has things in mind.
- There were quite a few questions, and it was considered whether it would be good to reduce them, for example, in the hospital ward when it comes to the child's well-being. A parent may not have the resources to respond to everyone.
- In the outpatient appointment time is short, is there time to respond? as a tool otherwise good.
- There was a lot of room for interpretation in some of the questions. Our group just considered the length of the survey. The checklist certainly makes the parent think things through, and yet it serves as good support for the discussion.
- For example, a family with few resources / multiple problems may not be able to focus on such a long survey
- The app needs some explanation of the rationale for its use and also what the anticipated outcomes would be from answering the questions.
- I would like more information on who would be using this with clients and what would be its purpose of it?
- I find it hard to imagine that one could get so much information by questioning the parents, especially on issues that they would recognize as being serious problems.
- certain questions related to the private lives of the parents and which most of these low threshold professionals would not be able to ask – it's not their business. It's other professionals (psychologists, care professionals) that might be able to use the checklist.
- we are unable to obtain, let alone assess, the sum of all those questions on the checklist. Even when working closely with families, gray areas remain legion.
- The app is very easy to use, uncluttered, focused and from a practitioner perspective, we like the way it highlights potential areas for focus/concern.
- The introductory paragraphs need some work on the turn of phrase, and an explanation of how the information will be used.
- Some countries will not be able to guarantee "seamless collaboration"….
- There are 55 questions, and even if you respond to these without thinking too much, that's a lot of questions...
- Some of the implications/wording of the questions might be perceived as leading/controversial
- Some questions may need rewording with the use of professional terminology
- We also wonder where it fits ie what evidence base/framework informs the questions are they based around the evidence of ACEs + wider public health areas? It feels a bit random as you go through them.
- Once you have the answers are there specific pathways recommended according to the responses?

- Maybe it is assumed that practitioners will have these pathways in place and that this app will be a tool to facilitate conversations between parents and practitioners?
- Overall we think it could be a good tool for practitioners in partnership with parents to highlight potential areas for support.
- We suggest grouping the questions; the parent's experience, pregnancy, and birth, the child's needs, the parent/child relationship, focus on parental public health knowledge
- What is the evidence base for these questions?
- Mobile application needs validation before official utilization
- Risk factors can vary in different cultures
