# Supplementary material for: Family Needs Checklist: Development of a Mobile Application for Parents with Children to Assess the Risk for Child Maltreatment
Source: Int J Environ Res Public Health. 2022 Aug 9;19(16):9810. doi: 10.3390/ijerph19169810 (PMC9408053; doi:10.3390/ijerph19169810)

Supplementary material

Figure S2: Outline of the modified checklist development process used in the development of the Family Needs Checklist. SME=subject matter expert, C1T= Cohort 1 Trainees, C2T= Cohort 2 Trainees, FNC= Family needs Checklist

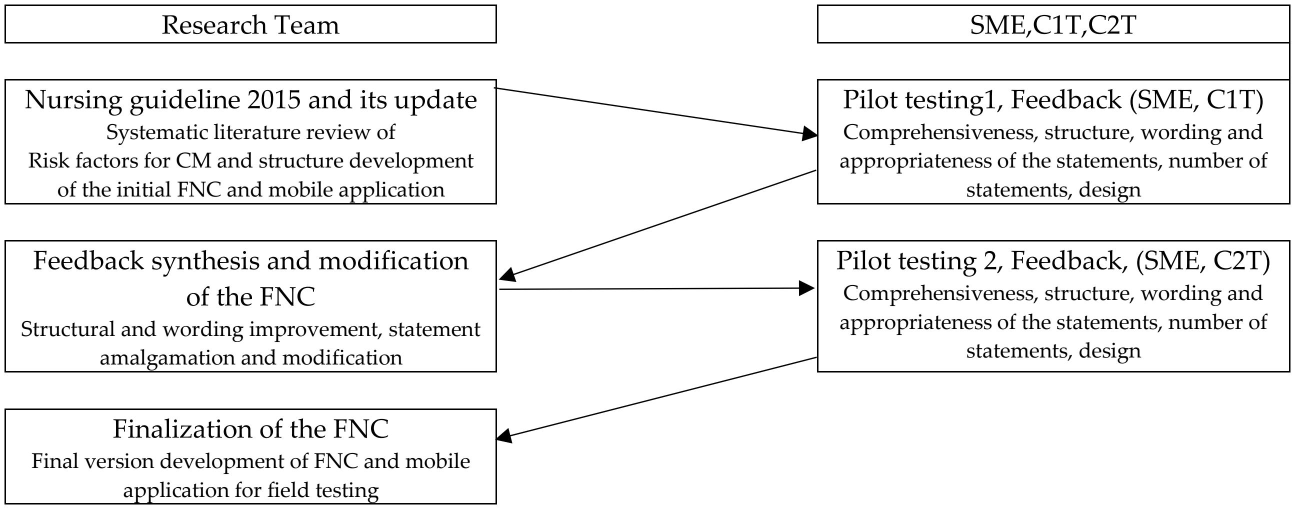

Supplement: Supplementary file 1 [file ijerph-19-09810-s001.zip › Supplementary material_Figure S2_Rantanen et al 2022 manuscript.pdf]
