# Supplementary material for: Family Needs Checklist: Development of a Mobile Application for Parents with Children to Assess the Risk for Child Maltreatment
Source: Int J Environ Res Public Health. 2022 Aug 9;19(16):9810. doi: 10.3390/ijerph19169810 (PMC9408053; doi:10.3390/ijerph19169810)

**Supplementary material Table S1**: Research questions, PICO and Search strategy Ovid Medline.

Research questions:

1. What are the factors related to the risk conditions of child maltreatment (CM)?
2. What instruments are reliable in identifying risk conditions in the family?

| **Research question** | **Population** | **Exposure of interest** | **Context/ Outcome** |
|---|---|---|---|
| Research question 1 | Child (0–17y) | CM risk or risk conditions specification or depiction | Specification or depiction of the CM risk or risk conditions in the child's family environment |
| Research question 2 | Child (0–17y) | Identification of CM risk or its risk conditions with a specified method or instrument | Identification of the CM risk or risk conditions |

| | |
|---|---|
| 1. | Child Abuse/ |
| 2. | Shaken Baby Syndrome/ |
| 3. | ((child$ or newborn$ or adolesc$ or teenage$ or infant$ or baby or babies or pediatr$ or paediatr$) adj5 (abus$ or maltreat$ or mistreat$ or neglect$ or assault$)).ti,ab,kf. |
| 4. | ((child$ or adolesc$ or teenage$ or infant$ or newborn$ or baby or babies or pediatr$ or paediatr$) adj5 ((corporal$ or physical$) adj3 punish$)).ti,ab,kf. |
| 5. | (Domestic Violence/ or Intimate Partner Violence/) and (child$ or newborn$ or adolesc$ or teenage$ or infant$ or baby or babies or pediatr$ or paediatr$).ti,ab,kf. |
| 6. | shaken baby syndrome.ti,ab,kf. |
| 7. | or/1-6 |
| 8. | diagnosis/ or exp "diagnostic techniques and procedures"/ or investigative techniques/ |
| 9. | Risk Assessment/ |
| 10. | exp psychiatric status rating scales/ or psychometrics/ |
| 11. | "surveys and questionnaires"/ or self report/ |
| 12. | Checklist/ |
| 13. | diagnostic self evaluation/ or self-assessment/ or self disclosure/ |
| 14. | Actuarial Analysis/ |
| 15. | (effect$ or valid$ or accura$ or utility or sensitiv$ or specific$ or observe$ or observat$ or incidence or quality).ti,ab,kf. |
| 16. | "reproducibility of results"/ |
| 17. | exp "Sensitivity and Specificity"/ |
| 18. | observer variation/ |
| 19. | risk.ti,ab,kf. |
| 20. | (psychometric$ or scale or scales or subscale$ or diagnos$ or screen$ or measure$ or evaluat$ or report$ or disclos$ or checklist$ or inventor$ or index or indices or instrument$ or tool or tools or questionnaire$ or inteview$ or assess$ or disclos$ or test or tests or identif$ or apprais$ or actuarial analys$ or evaluat$).ti,ab,kf. |
| 21. | (effect$ or valid$ or accura$ or reliabil$ or feasibil$ or feasible or precision or impact or utility or sensitiv$ or specific$ or observe$ or observat$ or incidence or quality).ti,ab,kf. |
| 22. | or/8-21 |
| 23. | 7 and 22 |
| 24. | "systematic review"/ |
| 25. | meta-analysis/ |
| 26. | ((systematic or umbrella or overview) adj3 review$).ti,kf. |
| 27. | (meta-analy$ or metaanaly$).ti,kf. |
| 28. | or/24-27 |
| 29. | 23 and 28 |
| 30. | limit 29 to yr="2014-current" |

**Figure S1**. Flowchart of the systematic review and meta-analysis selection process.

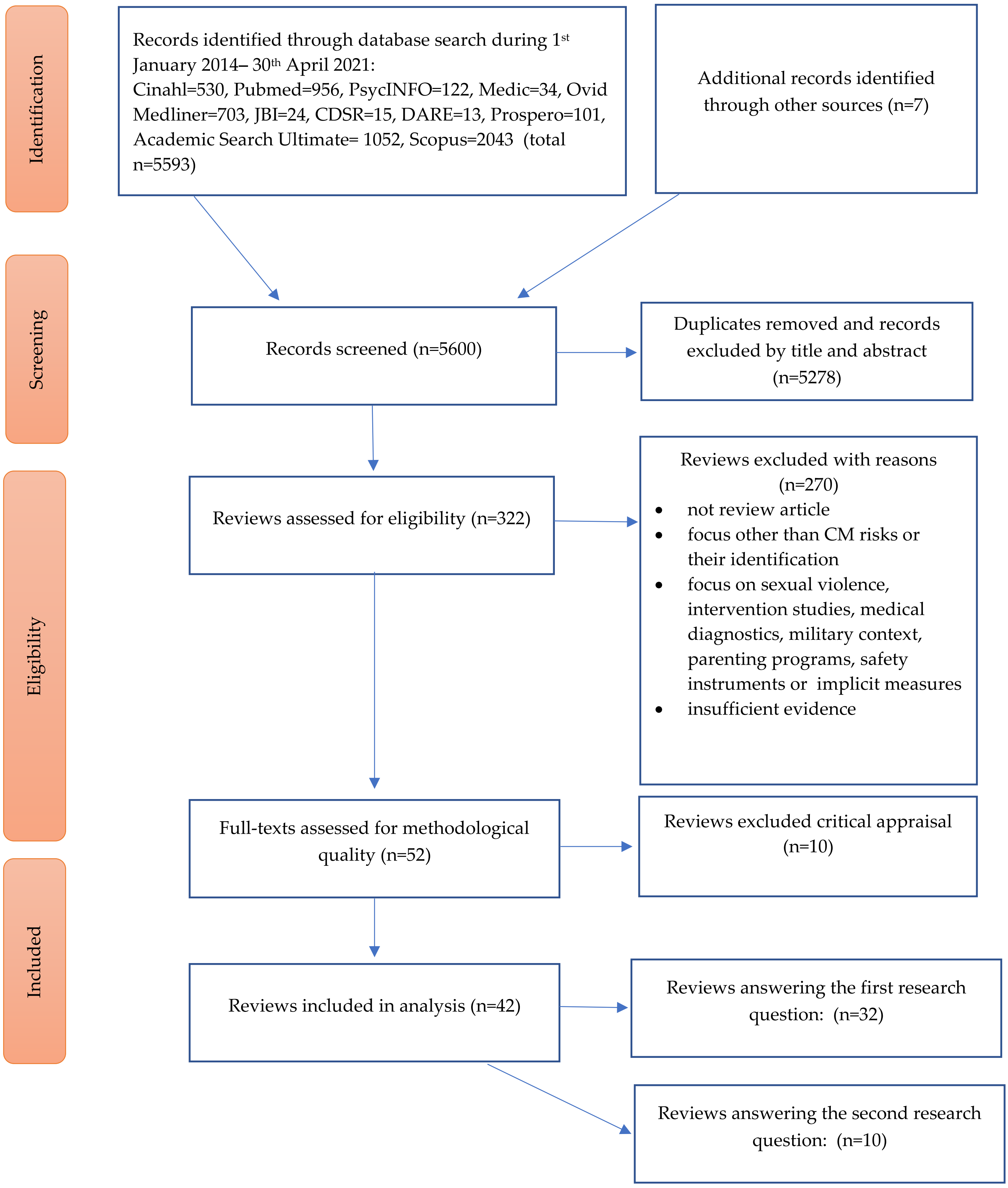

Supplement: Supplementary file 1 [file ijerph-19-09810-s001.zip › Supplementary material_Table S1 and Figure S 1_Rantanen et al 2022 manurcript.pdf]
