# Supplementary material for: Family Needs Checklist: Development of a Mobile Application for Parents with Children to Assess the Risk for Child Maltreatment
Source: Int J Environ Res Public Health. 2022 Aug 9;19(16):9810. doi: 10.3390/ijerph19169810 (PMC9408053; doi:10.3390/ijerph19169810)
Supplement: Supplementary file 1 [file ijerph-19-09810-s001.zip › Supplementary material_Table S2_Rantanen et al 2022 manuscript.pdf]

Supplementary material

Table S2. The report of the Family Needs Checklist (FNC) mobile application using the Qvalidi 2019® checklist [77].

**Item**	**No.**	**Description**	**Report of the Family Needs Checklist (FNC)**
**Basic details**			
Name	1	What is the name of the application?	Family Needs Checklist
Version	2	What version of the application is it?	version 1
Publisher and year of publication	3	Who published the application? When was the application published? Where can information be obtained about the publisher?	ERICA/ EU funded project/Paavilainen E (PI), Rantanen H & Research Team [30]
Developer	4	What organisation has developed the application? Where can information on the manufacturer be found in the application?	See above
Funding	5	Have significant external funders been involved in the development/publication of the application? If yes, who / which funding providers? Where can the user find information about the funder?	This research is part of the ERICA project funded by the European Union's Rights, Equality and Citizenship Programme (2014-2020). GA 856760.
Purpose	6a	What is the purpose of the application?	To help parents or caregivers to assess child maltreatment risks in the family context
	6b	Is the application a medical device?	No
	6c	How is the purpose of the application described to users in the location where the application is available?	It explained on the instruction page of the online application
Target group	7	For which target group is the application intended?	For parents and caregivers of the children under 18 years of age
Use	8	Where is the application intended to be used (in healthcare, independently during one's leisure time, elsewhere)?	Independently and contact services as per parent's need. Professionals can guide through the application if needed. The application itself includes instructions for use.

**Health Content**			
Health objective	9	What health or well-being objective is the application aimed towards?	Child maltreatment prevention in the family context
Background theory	10a	What background theory on health or well-being has been utilised in the modelling of the application and its operating principles?	1) WHO INSPIRE [1]<br>2) Preventing and Responding to Violence Against Children and Adolescents. A Theory of Change [46]<br>3) End Corporal Punishment, and end violence against children [22]
	11b	What is the basis for the background theory or thinking structure used in the application (theoretical literature, research evidence, expert experience)?	Clinical Practice Guideline 2022; *"Identifying conditions that may increase a risk of child maltreatment in a family"*,<br>which is based on multidisciplinary research evidence.[6]
Implementation of theory	12	How has the achievement of the health objective (and its underlying health theory/thinking structure) been implemented in the application?	Statements are based on international research evidence on child maltreatment risks. The parent or caregiver responds to the statements and receives feedback so that he or she can identify risk factors related to his or her own life, family life, or child and find support services that prevent child maltreatment in the family context.
Evidence	13	Is there research evidence on the health/well-being impacts of the application? Describe the key results and source data.	No, further testing is in progress
Health claims	14a	Does the application provide you with health or well-being information or contain claims about health/well-being?	Yes, claims about risk factors of the child maltreatment as well as protective factors
	14b	What are the claims and what evidence are these health claims based on (theoretical literature, research evidence, expert experience, other information)?	Clinical Practice Guideline 2022 *"Identifying conditions that may increase a risk of child maltreatment in a family"* [6,46]
Health information	15a	Does the application contain other health/well-being information (e.g. application uses information on the health effects of tobacco, but this information is not presented to the user in the form of a statement)?	No

	15b	What information does the application provide and what evidence is it based on (theoretical literature, research evidence, expert experience, other information)?	N/A
Consistency of recommendations	16	Is the health information contained in the application in line with current care recommendations?	Yes
Health sector expertise	17a	Has a health professional or expert (education, professional background) been involved or consulted in the application development process?	Yes
	17b	Has a professional or expert from the health sector or other suitable field assessed and approved the health content of the finished application?	Yes
**Technical properties**			
Technical specifications	18	With which platform or code language has the application been developed (e.g. Unity, HTML)?	Dart programming language and Flutter as a multiplatform framework
Application platform	19	Is the application native or browser-based? For native applications, how much memory does the download take up and does the application require internet access?	Both
Device	20a	What kinds of devices is the application intended for (mobile devices, desktops, laptops, other devices)?	Mobile devices, computers
	20b	If the application is available on mobile devices, which devices and operating systems are it compatible with?	WEB and Android
	20c	If the application is available for web browsers, which browsers are it compatible with?	The application can be used with any browser and with Android 19 and up
	20d	If the application is available on other devices, what are the specific details of these devices?	N/A
Accessories	21	Does the application require any additional hardware or software?	No

Availability	22	Where can the application be downloaded/accessed? Is there a charge for downloading or using the application?	Android→Play Store<br>WEB→ webpages.tuni.fi
Advertisements	23	Does the application contain advertisements? If yes, are the advertisements limited in some way?	No
Language	24	In which languages is the application available?	Finnish, English, Italian, German
Functionality	25	Provide a brief description of the application's functionality	Instruction page, information page, binary statements, results/feedback, and access to online services. Pdf-generation
Interaction	26	Does using the application involve interaction with other application users?	No
Time	27	How long does it take to use the application?	To be measured
Feedback	28	Does the application provide feedback to the user? If so, what kind of feedback? Is the feedback specific? What is the basis for the feedback?	Yes, evidence-based feedback on specific child maltreatment risk factors as well as protective factors to prevent child maltreatment in the family context.
Entertainment / gamification	29a	Is it a game or gamified application?	No
	29b	If it is a game or gamified application, what is the goal of the game?	N/A
	29c	Provide a brief description of the gaming / entertaining features and how the health/well-being content is linked to them.	N/A
Updates	30a	When was the application last updated? How often have updates been released?	30.11.2021<br>First time
	30b	How is the responsibility for updates specified in the event of errors being detected in the application?	The responsibility lies with the Tampere university
Integration	31	Can the application be integrated with other applications, devices, and/or patient information systems? If yes, what standard has been used?	No

Fill in items 32 to 35 only if the application is used in healthcare			
Patient/customer supervision	32a	Is the application intended to be used independently or together with a health care professional or other assistant (who)?	Both.<br>Can be used offline in a paper form together with parents or caregivers and the professionals of child and family health and social service. Certain legal restrictions must be adhered to, for example, restrictions on asking about the parents' or caregivers' criminal records which can be highly classified information.
	32b	What is the role of the healthcare professional in the use of the application?	The child and family service professionals should hold the expertise in the use of the Family Needs Checklist, child maltreatment knowledge, and support services.
	32c	Is a user manual available for healthcare professionals? Where can this manual be obtained? Is this manual freely available without charge?	Yes,<br>ERICA website [30]<br>Free of charge
Disinfection	33	Is it possible to disinfect the devices intended for use with the application?	N/A
Maintenance	34	What kind of maintenance is required for the devices used?	N/A
Testing	35	Has the application been tested in a healthcare environment? If so, how and when was the testing carried out, and what were the key results?	No. The testing is in progress.

**User-orientation**			
Ease-of-use	36a	Has the application's ease-of-use been assessed? What were the key results of the usability testing?	Only a preliminary assessment is done, and further testing is in progress
	36b	Have the intended end users of the application been involved in the development of the application?	Not yet. The development is in progress.
	36c	Has feedback on the finished application been collected from end users?	Not yet. The development is in progress.
Understandability	37	Has the understandability of the application and the information and feedback it provides been assessed from the end users' perspective? What were the key results of this assessment?	Not yet. The development is in progress.

User manual	38a	Is a user manual available for the application and where can it be obtained?	There is an instruction page available in the online application for the users and professional and training manual available for the professionals [30]
	38b	If a manual is available, is the application's intended use indicated in the manual?	Yes
Training	39	Is there training involved in the deployment of the application? If yes, who is the training intended for and how has it been implemented?	Training is needed for the professionals and is available through ERICA webpages [30].

Accessibility	40	Does using the application require specific skills or expertise? Does the application comply with the EU Accessibility Directive (2016/2102)? Is it possible for the user to give feedback on the application (how)?	No. The online application requires reading skills and internet skills. The application can be used offline as a paper form only together with the professional. In that case, there is no online feedback option. Support service options can be delivered through the professional. It must be noted, that with offline use, certain legal restrictions must be adhered to, for example, restrictions on asking about the parents' or caregivers' criminal records which can be highly classified information. The option of a self-referral offline version will be taken into account in subsequent projects.
Appearance	41	Is the application's appearance (layout, graphics) clear and attractive?	Yes, feedback is obtained from the professional perspective. The user perspective and feedback process is in progress.
**Safety**			
Technical reliability	42a	Has the technical reliability of the application (e.g. reliability of the measurement results collected by the application) been tested with different devices and operating systems?	Yes
	42b	Has the technical reliability of the application been tested under different conditions?	Not yet. The development is in progress.
Safety	43a	From the user's perspective, are there potential risks associated with using the application? How have the risks been identified?	Questions in this app may cause parental anxiety or self-inflicted trauma. The application provides links to various support services, even in urgent situations.
	43b	If there are potential risks, is the user warned of these?	Not on the instruction page but yes, in the feedback section

Age limit recommendation	44	Is there an age limit recommendation for the application?	No
Privacy	45a	Does the use of the application require login? If yes, how is the user identified?	No
	45b	Has the development of the application been carried out in compliance with the General Data Protection Regulation (GDPR) and the EU Code of Conduct on Health App Privacy?	Yes, Available in the Google Play store free of charge. The privacy policy information is available for users through the instruction page https://projects.tuni.fi/erica/mobile-app/privacy-policy/
Data storage and confidentiality	46a	Does the application collect or store data about the user? (including cookie policy, passwords, and other user information)	No, only the amount of users can be detected by creators. There is a privacy policy explained by the third-party service providers: Google Play Services, Google Analytics for Firebase, and Firebase Crashlytics. https://projects.tuni.fi/erica/mobile-app/privacy-policy/
	46b	If yes, what information is collected and what is the collected information used for?	N/A
	46c	Where is the data stored and who owns it?	N/A
	46d	How is the data storage and collection indicated to the user and where is the data available?	N/A
Informing data subjects	47	Is the user informed about the processing of their personal data?	N/A
Data security	48a	If the application collects or stores data about the user, how is the data protected?	N/A
	48b	What measures are taken to protect against data security attacks?	N/A
Ethics	49	How has the ethical conformity of the application been ensured?	The content is based on systematically searched and analysed multidisciplinary research evidence that is in line with both international and national action programs [1, 29,46, 28] It is also in line with the Foundation's action programs to reduce and prevent violence or abuse against children and to promote the health and well-being of children [22] as well as

			with related national and international laws and regulations concerning child maltreatment prevention [111–114]. Questions in this app may cause parental anxiety or self-inflicted trauma. Therefore, the application provides links to various support services, even in urgent situations.

* It is recommended to monitor for updates to checklists and to use the most recently published checklist
