# Supplementary material for: Family Needs Checklist: Development of a Mobile Application for Parents with Children to Assess the Risk for Child Maltreatment
Source: Int J Environ Res Public Health. 2022 Aug 9;19(16):9810. doi: 10.3390/ijerph19169810 (PMC9408053; doi:10.3390/ijerph19169810)
Supplement: Supplementary file 1 [file ijerph-19-09810-s001.zip › Supplementary material_Table S3_Rantanen et al 2022 manuscript.pdf]

Supplementary material

Table S3. Reviews included in the evidence extraction process.

**Authors, Year, Country, [reference number] and The countries of the original studies**	**Study objective and participants**	**Research methods and designs of the original studies**	**Key findings**	***Level of evidence **Critical appraisal score**
Madigan S, Cyr C, Eirich R, et al. 2019. Canada. [11]<br><br>Studies were conducted in the United States (n = 94), Europe (n = 26), South America (n = 7), Australia and New Zealand (n = 7), Asia and Africa (n = 8).	Intergenerational transmission of child maltreatment in families.<br><br>The sample size ranged from 25 to 85,084 (Median n= 222). The participants were mothers (n = 39 studies), fathers (n = 19 studies), parents whose gender was not stated (n = 44 studies), relatives (n = 8 studies) and perpetrators who were not further specified (n = 34 studies).	Systematic review and meta-analysis (n = 142 studies)<br><br>epidemiological cross-sectional studies<br>intervention studies<br>longitudinal studies<br>prospective cohorts<br>retrospective cross-sectional studies.	**Parents who have been subjected to maltreatment in childhood are at an increased risk of maltreating their own children.** A moderate association was found between parental history of maltreatment history and child maltreatment in the next generation d = 0.45, 95% CI [0.37, 0.54], n = 80 studies.	**A***<br>**11/11****
Savage L, Tarabulsy G, Pearson J, et al. 2019. Canada. [13]<br><br>The studies were conducted in the United States (n = 19), Canada (n = 7), Germany (n = 2), the Netherlands (n = 2), the United Kingdom (n = 1), and Australia (n = 1).	Maternal history of childhood maltreatment and later parenting<br><br>A total of 17,932 participants were mothers of 0–6-year-old children. The sample size ranged from 35 to 8,292 participants.	Systematic review and meta-analysis (n = 32 studies)<br><br>The research designs of the original studies: N/A	**Parents who have been subjected to maltreatment in childhood are at an increased risk of maltreating their own children**<br>In 27/32 studies, a connection was found between a parent's childhood maltreatment experience and the parent's maltreatment behavior directed at their own child (r = -0.13 (95% CI [-0.17, -0.09] p < 0.001). The research supports the hypothesis that a parent's childhood experiences of maltreatment can be considered a potential risk for parents' later abusive or negative behavior towards their own children.	**A***<br>**10/11****
Assink M, Spruit A, Schuts M, et al. 2018. [78]<br><br>Studies were conducted in the United States or Canada (n = 71), Europe (n = 12) and Australia (n = 1)	Intergenerational transmission of child maltreatment<br><br>Parents	Systematic review and meta-analysis (n = 84 studies / 285 effect sizes)<br>prospective studies (n = 25)<br>retrospective studies (n = 60)<br>cross-sectional studies (n = 22)<br>longitudinal studies (n = 62)	**Parents who have been subjected to maltreatment in childhood are at an increased risk of maltreating their own children**<br>Parents who experienced maltreatment in their childhood had about three times the risk of maltreatment of their own children (OR = 2.990; medium summary effect: r = 0.289 95% CI [0.257, 0.337] p < 0.001).	***A**<br>**11/11****
Mulder T, Kuiper K, van der Put C, et al. 2018. The Netherlands. [12]	Risk factors for child neglect.	Systematic review and meta-analysis	**Many risk factors related to the family increase the risk of child maltreatment. Parents who have been subjected to maltreatment in childhood appear to be at an increased risk**	**B***<br>**9/11****

The studies were conducted in the United States (n = 33), South Korea (n = 1), Vietnam (n = 1), and the Netherlands (n = 1).	There were a total of 729,840 children examined, of which 19,851 were victims of neglect.<br><br>The sample size ranged from 23 to 495,368.	retrospective/RC studies (n = 27) prospective studies (n = 9)	**of neglecting their own children.** (mean r = 0.182 95% CI 0.108–0.259; p < 0.001)<br><br>**Parent's antisocial and criminal background, young age and low level of education appear to be risk factors for child neglect.**<br><br>Parent-related risk factors were the parent's antisocial personality and criminal background (mean r = 0.372; 95% CI [0.168, 0.615] p = 0.011), young age (< 20 years) (mean r = 0.140; 95% CI [0.065, 0.217] p = 0.001) and low level of education (mean r = 0.229; 95% CI [0.147, 0.318] p < 0.001).<br><br>**Parents' mental health issues or physical problems appear to increase the risk of child neglect.**<br><br>Parent-related risk factors for child neglect were the parent's previous mental health problems (mean r = 0.259; 95% CI [0.118, 0.411] p = 0.002) and mental health or physical problems (mean r = 0.207; 95% CI [0.063, 0.357] p = 0.007).<br><br>**Problems during the perinatal period, the child's behavioural problems and physical problems appear to be risk factors for child maltreatment.**	
Li S, Zhao F, Yu G. 2020. China. [15]<br><br>Origin of studies: N/A	Childhood maltreatment of and intimate partner perpetration<br><br>The number of participants varied between studies (n = 32,544)	Systematic review and meta-analysis (n = 63 studies / 87 effect sizes<br><br>prospective cross-sectional studies(n = 15 effect sizes) retrospective cross-sectional studies (n = 72 effect sizes)	**Maltreatment experienced by parents in childhood appears to increase their risk of intimate partner violence.**<br><br>Maltreatment experienced in the childhood (physical, emotional or sexual) was associated with later perpetration of intimate partner violence (r = 0.16, 95% CI [0.14, 0.18] p < 0.001). In subgroup comparisons, physical maltreatment experienced in childhood (r = 0.17, 95% CI [0.14, 0.20] p < 0.001), emotional maltreatment (r = 0.13, 95% CI [0.07, 0.19] p < 0.001) and sexual violence (r = 0.13, 95% CI [0.07, 0.18] p < 0.001) were related to later perpetration of intimate partner violence. There was too little research material on childhood neglect to carry out a meta-analysis. For men, the association was stronger (r = 0.20, 95% CI [0.16, 0.24]) compared to women (r = 0.11; 95% CI [0.09, 0.14]). No significant differences in the results were observed between marital status or between the measures measuring childhood maltreatment.	**B***<br><br>**10/11****
Lo C, Chan K, Ip P. 2019. Hong Kong. [14]	Insecure adult attachment and child maltreatment	Systematic review and meta-analysis (n = 16 studies)	**Parents' experiences of an insecure attachment style in their childhood increase their child's risk of maltreatment.**<br><br>87.5% of abusive parents had an insecure attachment relationship compared to non-abusive parents (64.5%) and the	**A***<br><br>**10/11****

The studies were conducted in the United States, Canada and Italy.	The sample size varied between 48 and 276 participants. A total of n = 1,830 parents participated in the research, most of whom were mothers. One study focused on both mothers and fathers and one study focused only on fathers.	cross-sectional studies.	general population (43%). Unresolved attachment relationship was overrepresented in the group of abusive parents (46.4%).<br><br>Compared to non-abusive parents (23.4%) and the general population (14.8%). Parents with an insecure attachment relationship with their own parent (OR = 2.63; 95% CI [1.81, 3.83] p = 0.000), their partner (OR = 3.76; 95% CI 2.03, 6.00] p = 0.000), to their own child (OR = 3.13; 95% CI [1.55, 6.33] p = 0.001), or in general (OR = 3.38; 95% CI [2.14, 5.32] p = 0.000) an association with maltreatment of one's own children was observed, regardless of the object of the attachment relationship.<br><br>Parents who experienced an insecure attachment relationship had an increased risk of maltreatment of their own child (pooled effect size: OR = 2.93; 95% CI [2.31, 3.72] p = 0.000). In outcome variable-specific subgroup analyses, an insecure attachment relationship was associated with the risk of maltreatment of one's own children (OR = 3.52; 95% CI [2.65, 4.66] p = 0.000).	
Montgomery E, Just-Østergaard E, Jervelund S. 2019. Denmark. [84]<br><br>The studies were conducted in Sri Lanka (n = 2), Croatia (n = 1), Palestine (n = 2), Rwanda (n = 1), Uganda (n = 1), the United States (n = 5), China (n = 1) and the Netherlands (n = 2).	The risk of child abuse perpetrated by parents exposed to traumatic events<br><br>The sample size varied from 92 to 4,327 participants.	Systematic review (n = 15 studies)<br><br>cross-sectional studies (n = 11) case-control studies (n = 4)	**Trauma experienced by parents appears to increase their child's likelihood of being subjected to maltreatment.**<br><br>In the groups of parents exposed to traumatic events, the prevalence of child abuse was 36–97.5%, regardless of the type of trauma (natural disaster, war, genocide or serious political violence). The prevalence of severe physical abuse of the child was 2.5–18% in the groups of parents exposed to trauma. The severity of the trauma was related to the maltreatment of the parents towards their children in most studies (β = 0.13–0.17; OR = 1.66). Regardless of the type of traumatic event, parents with a diagnosis of post-traumatic stress disorder had a greater risk of moderate physical abuse (OR = 1.38) and severe physical abuse (OR = 1.93) than parents without this diagnosis (β = 0.03–0.15). Mothers who experienced sexual violence were found to have an increased risk of severe physical abuse of their child (OR = 1.66; $p < 0.05$). Exposure severity was associated with maternal neglect, reported maltreatment, and severe physical discipline. War trauma experience had a significant relationship with fathers' increased emotional abuse of children (r = 0.17 and 0.04; $p < 0.05$). Of the children	**B***<br><br>**11/11****

			who participated in the study, 82.1% reported being exposed to emotional abuse.	
Plant D, Pawlby S, Pariante C, et al. 2018. UK. [82]<br><br>The studies were conducted in the United States (n = 5), the United Kingdom (n = 3), Spain (n = 2), Norway (n = 2), and the Netherlands (n = 1)	Maternal childhood trauma and offspring child psychopathology<br><br>The sample size varied between 31 and 25,452 participants). Total of 45,723 participants	Systematic review (n = 12 studies) descriptive analysis<br><br>Longitudinal studies (n = 10), with (n = 10) cohorts and (n = 2) cross-sectional studies.	**The children of mothers who have been subjected to maltreatment in childhood appear to be at a higher risk of behavioural problems and mental disorders.**<br><br>A positive connection was found between the mother's childhood maltreatment and the children's internalising and externalising behavior problems and clinical mental health disorders. In all the studies measuring the child's problems, at least one positive connection between the mother's childhood abuse and the child's behavior problem was reported. Two studies found a connection between the child's externalizing problems and the maltreatment experienced by the mother in childhood (rb = 0.11) and emotional maltreatment (B = 0.6; 95% CI [0.4, 0.7]) and physical or sexual violence (B = 0.4; 95% CI [0.3, 0.6]). One study found a connection between a child's internalising problems and the mother's childhood abuse (β = 0.04). When examining the connection between the child's behavior disorders and the victimization experienced by the mother in her childhood, four studies found a connection with both internalising and externalising problems.	**B***<br><br>**9/11****
Su Y, D'Arcy C, Meng X. 2022. Canada. [83]<br><br>The studies were conducted in the United States (n = 5), the United Kingdom (n = 3), China (n = 1), Canada (n = 2), and the Netherlands (n = 1).	Effect of maternal childhood maltreatment on next generation's vulnerability to psychopathology<br><br>The sample size of mother-child pairs varied from 96 to 11,402. Total n = 29,682 participants.	Systematic review and meta-analysis (n = 12 studies)<br><br>prospective cohort studies	**The children of mothers who have been subjected to maltreatment in childhood appear to be at a higher risk of behavioural problems and mental disorders[40,41]. (B)**<br><br>A weak association (r = 0.12; 95% CI [0.11, 0.14]) was found in 12 studies between mothers' childhood maltreatment experiences and their children's psychopathology. In four studies, the association was moderate (r = 0.30).	**B***<br><br>**11/11****
Langevin R, Marshall C, Kingsland E. 2021. Canada. [16]<br><br>The studies were conducted in the United States, the United Kingdom, Canada, Mexico and China.	Intergenerational cycles of maltreatment. Psycho-social risk and protective factors<br><br>The sample size of mother-child pairs/mothers/fathers/children varied from 9 to 11,384. Total n= 22,728	Systematic review (n = 51 studies of which n= 33 providing data on risk and protective factors)<br><br>longitudinal studies<br>cross-sectional studies<br>cohort studies<br>secondary analysis of data collected for purposes other than studying intergenerational maltreatment	**Both risk factors and protective factors for intergenerational maltreatment have been identified.**<br><br>Risk and protective factors were described from the perspectives of the parent's individual characteristics (26/34 studies), factors related to interpersonal relationships (29/32 studies) and factors related to circumstances (6/6 studies).<br><br>Significant risk factors related to the individual characteristics of the parent were the mother's mental health disorder (3/5 studies), PTSD symptoms (1/1 study), anxiety (3/3 studies-black), depression (3/4 studies) and substance use (6/8 studies), mother's young age (4/6 studies), adverse childhood	**A***<br><br>**8/11****

			experiences (7/8 studies), mother's aggressive reaction tendency (1/1 study), mother's authoritarian parenting attitude (1/1 study), violence defensive authoritarian attitude (1/1 study), experience of justifying maltreatment (1/1 study), antisocial behavior (1/1 study), mother's experience of sexual violence (1/1 study), several forms of maltreatment experienced by the mother in her childhood (1/ 1 study), maltreatment experienced in youth (1/1 study), witnessing intimate partner violence in childhood (1/1 study), insecure attachment relationship experienced by the mother during childhood (1/1 study) and low number of years lived with own mother (1 /1 study).<br>Significant relationship-related risk factors were intimate partner violence experienced by the mother (7/7 studies), relationship problems (1/1 study), social isolation and lack of support (3/5 studies), parenting problems (2/3 studies), use of disciplinary violence (1/ 1 study), insufficient supervision of children (1/1 study), authoritarian parenting style (1/1 study) and parenting stress (1/1 study).<br>Significant risk factors related to circumstances were living with a violent adult or partner with a criminal background (2/2 studies), children's exposure to community abuse (1/1 study), stressful life events (1/1 study) and financial stress (2/2 studies).<br>Protective factors related to the individual characteristics of the parent were the mother's participation in therapy (1/1 study) and the parent's ability to self-control (1/1 study). Protective factors related to human relationships were safe, stable, supportive and nurturing relationships in the family and in a relationship (3/5 studies), secure attachment to the child and maternal warmth (2/2 studies) and satisfaction with one's parenting (1/1 study) . The protective factor related to the circumstances was the family's higher socioeconomic status (2/2 studies).	
Chamberlain C, Gee G, Harfield S, et al. 2019. Australia. [17]<br><br>The studies were conducted in the United States, Australia, Canada,	Parenting after a history of childhood maltreatment in the perinatal period<br><br>Participants were mothers, mothers and fathers and mothers or fathers.	Systematic review (n = 74 articles / n = 57 studies), among other things, risk factors and protective factors for intergenerational child maltreatment were investigated (n = 38 studies).<br><br>intervention RCT studies (n = 2)<br>RCT studies (n = 1)<br>RCT/SEM studies (n = 1)	**Both risk factors and protective factors for intergenerational maltreatment have been identified.**<br>The studies found risk factors stemming from childhood that led to transgenerational maltreatment. These included deprivation or poverty, young age, social isolation or poor social functioning, use of drugs or alcohol, smoking, intimate partner violence, lack of knowledge about child development, poor mental health, stress, symptoms of depression, challenges in expressing emotions, post-traumatic stress	**A***<br>**9/11****

Germany, the Netherlands and the United Kingdom		cross-sectional studies including RCT design (n = 1)<br>prospective cohorts (n = 17)<br>cross-sectional studies (n = 11)<br>cross-sectional studies from a longitudinal study cohort (n = 4)<br>multi-method studies (n = 3)<br>longitudinal studies (n = 6)<br>qualitative interview study (n = 7)<br>case studies (n = 4).	symptoms or dissociation disorder. These are connected to the parent's weakened confidence in their own parenting abilities when the baby is crying, sleep problems, parenting stress, negative interpretations of the baby, bad parenting practices, weak parental warmth, negative coping strategies such as excessive sleeping, eating or working, and valuing disciplinary violence. These were further linked to the baby's inheritance, low birth weight, premature birth, insecure attachment and difficulty forming close relationships, victimization and poor social and emotional development of the baby.<br>The research found protective factors against the effects of intergenerational abuse, such as parental persistence or tolerance, financial solvency, access to services, support from family and social circle, warmth and positive relationships in a relationship, belief in managing events that affect oneself, ability to be flexible, self-care, participation in counseling, meditation, volunteer work, exercise, mentalization (the ability to think about one's own and another person's perspective and experience), developing an attachment relationship, and parenting skills training. These were related to more positive parent interaction, satisfaction with own parenting, positive attitudes toward parenting, perceptions of improved parenting, secure attachment, reduced harsh parenting, and reduced infant maltreatment.	
Aho A, Remahl A, Paavilainen E. 2017. Finland. [88]<br><br>Origin of studies: N/A	Homicide in the western family and background factors of a perpetrator	Systematic review n= 32 studies<br><br>The material consisted of national register materials of different countries, hospital materials and qualitative material.	**Many factors, such as the parent's stress, difficulties in social relationships, violent behaviour and crime appear to be risk factors for familicide.**<br>**Parents' substance use appears to be a risk factor for familicide.**<br>**Parents' mental health issues appear to increase the risk of familicide.**	**B***<br>**10/11****
Hyysalo N, Gastelle M, Flykt M. 2021. Finland and USA. [91]<br><br>The studies were conducted in the United States.	Maternal pre- and postnatal substance use and attachment in young children<br>A total of 1,841 mothers were examined. The sample size varied from 42 to 860. The age of the	Systematic review and meta-analysis (n = 11 studies)<br><br>cross-sectional studies (n = 5)<br>longitudinal studies (n = 6)	**Substance abuse by mothers during pregnancy and after delivery appears to be related to an insecure attachment relationship experienced by the child.**<br>An association was found between the mother's substance use during pregnancy and postpartum (12–57 months) (n = 1,841)	**B***<br>**11/11****

	children was most often 12–24 months (max. 57 months)		and the child's insecure attachment relationship (pooled effect size r = -0.10; 95% CI -0.18–-0.02; p = 0.018).	
Kuppens S, Moore S, Gross V, et al. 2020. Belgium and UK. [92]<br><br>The studies were conducted in the United States (n = 35), Europe (n = 9), Australia (n = 9), Asia (n = 2), and South America (n = 1).	The enduring effects of parental substance use on child's well-being<br><br>Participants were parents (n = 79), mothers (n = 59), fathers (n = 82) and children aged 0–18 years (n = 218).	Systematic review and meta-analysis (n = 56 studies/220 effect sizes = t)<br><br>observational longitudinal studies	**Parents' substance abuse appears to be related to the child's reduced well-being.**<br><br>An association was found between parental substance abuse and poorer child well-being (r = 0.15; 95% CI [0.12, 0.17] t (219) = 12.94; p < 0.001). Comparisons showed that the association of children's poor well-being with parental drug use was stronger when comparing well-being with alcohol use (t (205) = 3.75; p < 0.001), smoking (t (205) = 3.66; p < 0.001) or to alcoholism (t (205) = 2.83; p = 0.005). In five studies (n = 10 effect sizes), of the drugs used by parents, especially cannabis (71%) was identified as a significant factor in children's poorer well-being (r = 0.23; 95% CI [0.14, 0.32] p < 0.001).	**B***<br><br>**10/11****
Cafferky BM, Mendez M, Anderson JR, et al. 2018. USA. [90]<br><br>The studies were conducted in the United States (n = 204), Albania (n = 1), Australia (n = 3), Bolivia (n = 1), Brazil (n = 1), Cambodia (n = 1), Canada (n = 19), China (n = 2), Dominican Republic (n = 1), Ethiopia (n = 2), Haiti (n = 1), Netherlands (n = 1), Hong Kong (n = 2), India (n = 5), Indonesia (n = 1), Iraq (n = 1) Japan (n = 1), Jordan (n = 1), Kenya (n = 1), Malawi (n = 1), Mexico (n = 1), Mozambique (n = 1), Myanmar (n = 1), New Zealand (n = 5), Nicaragua (n = 1), Nigeria (n = 3), Norway (n = 2), Peru (n = 2), Philippines (n = 1), Puerto Rico (n = 1), Rwanda (n = 1), South Africa (n = 6), Spain (n = 1), Sri Lanka (n = 1), Sweden (n = 2), Tanzania (n = 1), Thailand (n = 1), Turkey (n = 2), Uganda (n = 1) and Ukraine (n = 1)	Substance use and intimate partner violence<br><br>The sample size varied between < 100 and > 30,000 (n = 627,726). 73% of the studies had < 1000 women and men participants.	Meta-analysis (n = 285 studies and n = 983 effect sizes = ES), k = amount of studies<br>Quantitative studies	**Parents' substance abuse appears to be related to the physical intimate partner violence.**<br><br>A relationship was found between substance use and partner violence (r = 0.22; SE = 0.005; 95% CI [0.20, 0.24] p < 0.001; k = 215). Similarly, a relationship was found between substance use and being a victim of intimate partner violence (r = 0.20; SE = 0.004; 95% CI [0.18, 0.22] p < 0.001; k = 146). When comparing women and men, differences were found between the use of substances and the perpetration of physical violence. A stronger relationship was observed between men's use of substances and committing violence (r = 0.23; p < 0.001; k = 399) compared to women using substances (r = 0.17; p < 0.001; k = 109). A stronger connection was observed between men's alcohol use and committing violence (r = 0.22; p < 0.001; k = 277) compared to women who use alcohol (r = 0.15; p < 0.001; k = 77).	**B***<br><br>**9/11****
Frederick J, Devaney J, Alisic E. 2019. UK and Australia. [89]	Homicides and maltreatment-related deaths of disabled children	Systematic review (n = 25 studies)	**An inability of the parents to respond to the child's special needs and a lack of understanding of the special needs**	**B*/C***<br>**8/11****

The studies were conducted in the United States (n = 10), the United Kingdom (n = 4), Australia (n = 2), Italy (n = 2), Sweden (n = 2), Belgium (n = 1), Canada (n = 1), in China (n = 1) and in Finland (n = 1)	The sample size varied from 1 to 202465.	case studies (n = 1)<br>case series (n = 21)<br>epidemiological studies (n = 3)	**appear to increase the risk of neglect that could lead to filicide. (B)**<br>In four studies dealing with child deaths (n = 7,736), the death of a special child (n = 36) was caused by neglect of the child's basic needs or intentional neglect of treatment and care with the intention of causing the child's death (deprivational abuse). The parents of a special child showed a lack of understanding of the child's special needs or an inability to meet these special needs.<br><br>**Disability or autism in children may increase the risk of filicide. (C)**	
Bhatia S, Maguire S, Chadwick B, et al. 2014. UK. [98]<br><br>The studies were conducted in the United States (n = 5), Australia (n = 1), Italy (n = 1) and Sweden (n = 1)	Characteristics of child dental neglect<br><br>The sample size varied between studies. There were a total of participants (n = 1,595 children).	Systematic review (n = 9 studies)<br><br>case-control studies (n = 2)<br>case studies and case series (n = 4)<br>retrospective cohorts (n = 2)<br>cross-sectional studies (n = 1)	**Neglecting children's dental care appears to cause significant, long-term harm to children.**<br>Neglect caused harmful effects to the child, such as caries, plaque, oral infections, bleeding of the oral mucosa, loose teeth, pain, swelling, difficulty biting and avoidance of smiling.	**B***<br>**8/11****
Bradbury-Jones C, Isham L, Morris A, et al. 2021. UK. [96]<br><br>The studies were conducted in West Asia, India, South America, Europe, Africa, North America and Australia (n = 23 countries)	The connection between child maltreatment and oral health<br><br>Participants were children	Scoping review (n = 69 articles) / (n = 57 studies)<br><br>descriptive studies such as case-control studies, retrospective cohorts, cross-sectional studies and clinical evaluations. In addition, eight of the studies were reviews, two qualitative studies and one mixed-method study.	**Poor oral health in children may be related to child maltreatment.**<br>In seven studies, children who experienced maltreatment were compared with the normal population. Children who experienced abuse were found to have poorer oral health than children in the control group. Poor oral health manifested itself as caries, missing first teeth, permanent dental decay due to neglect, stained teeth and missing appointments. Five studies investigated the characteristics of head and neck injuries in children who had experienced maltreatment.	**C***<br>**8/11****
The Royal College of Paediatrics and Child Health - RCPCH. 2017. UK. [97]<br><br>Origin of studies: N/A	Child protection evidence -dental neglect<br><br>Participants were children	systematic review (n = 37 articles / 31 studies)<br><br>prospective comparative studies (n = 2)<br>case studies (n = 29).	**Unexplained oral injuries appears to be a sign of child maltreatment.**<br>11% of oral lip tendon injuries were caused by severe abuse of the child. A child's torn lip tendon alone was not found to be a sign of child abuse.	**B***<br>**9/11****
Sylvestre A, ÈL B, Bouchard C. 2016. Canada. [99]	Language problems among abused and neglected children	Meta-analysis (n = 22 studies / 23 samples)	**Child neglect or physical maltreatment may be linked to a delay in the child's language development.**<br>The language development of physically abused and/or neglected children was more delayed than that of children	**C***<br>**10/11****

The studies were conducted in the United States (n = 10), Canada (n = 2) and England (n = 1)	The sample size varied between studies (n = 24–142). A total of 1,420 children	cohort studies with group comparisons	who had not experienced abuse and/or neglect (g = - 0.53; p < 0.001; 95% CI [-0.71, -0.36]) (After the trim-and-fill procedure, g = -0.45). In the subgroup analysis, physical abuse, neglect, and physical abuse and neglect were all connected to the delay in children's language development (Q' = 1.30; p = 0.52), and the connection was equally large in all subgroups. In addition, the child's age had a significant effect on the connection between neglect and/or other maltreatment and language skills. In younger children, the negative effect turned out to be greater (slope = 0.06; p < 0.01) than in older children.	
Ayers S, Bond R, Webb R, et al. 2019. UK. [94]<br><br>The studies were conducted in the United States (n = 14), Great Britain (n = 3), Japan (n = 3), India (n = 1), Australia (n = 1), South Korea (n = 1) and New Zealand (n = 1)	Perinatal mental health and risk of child maltreatment<br><br>The sample size varied from 48 to 14,893. The majority of participants belonged to low-risk parents (n = 17 studies). In seven studies, the subjects were high-risk parents.<br>The majority of studies (n = 17) examined mothers and young children, one examined pregnant women, four examined both mothers and fathers, and two examined only fathers.	Systematic review and meta-analysis (n = 24 studies / 30 articles)<br><br>cross-sectional studies (n = 15)<br>longitudinal studies (n = 5)<br>cohort studies (n = 4).<br>The studies in which the connection had been studied statistically were accepted for the meta-analysis (n = 17 studies / 22,042 parents).	**Any mental health issues in parents during pregnancy and 12 months following childbirth appear to be related to an increased risk of child maltreatment, especially when the mental health issue is severe or there are other, simultaneous risk factors for child maltreatment in the family.**<br><br>61.3% of the studies (n = 19) supported the connection of mental health problems in the parent during the perinatal phase (pregnancy and 12 months after birth) with an increased risk of child maltreatment. A meta-analysis showed that poor perinatal mental health of the parent was associated with an increased risk of child maltreatment (OR 3.04; 95% CI 2.29–4.03). The risk was higher for mothers belonging to the risk group who had a serious mental health problem, such as severe depression or psychotic beliefs, or other risk factors, such as violence in the family. Studies examining fathers (n = 6) found a connection between mental health problems such as depression and child abuse. In addition, the low presence of fathers in family life and, on the other hand, the psychological abuse, coercion or violence used by fathers towards the mother increased the risk of child abuse.	**B***<br><br>**10/11****
Boorman R, Creedy D, Fenwick J, et al. 2019. Australia. [87]<br><br>The studies were conducted in Finland, the USA, Canada and Germany	The review examined the empathy of pregnant mothers and first-time mothers.<br><br>In almost all studies, only mothers were studied.	Systematic review (n = 13 studies)<br><br>cross-sectional studies<br>longitudinal studies, four of which included a control group.	**Severe anxiety during pregnancy and in the postpartum period appears to impair the mother's ability to emphasise with the child and increase the mother's feelings of frustration and thoughts related to harming the child.**<br><br>In a study measuring the relationship between a child's prolonged (10 min.) crying and the mother's thoughts about harming the child, as well as the proportions of negative mood, irritability and empathy, the mother's higher affective empathy, i.e. the ability to feel another person's feelings and respond to them, predicted fewer thoughts about harming the	**B***<br><br>**9/11****

			child (xb = 22 .43; p < .001) than the mother's lower affective empathy ability. When stressed, personal anxiety superseded affective empathy (xb = 37.16; $p < 0.001$). In a study that examined the relationship between maternal frustration and prolonged infant crying, the mother's lower cognitive empathy, i.e. the ability to understand another person's feelings ($r = -0.30$; $p = 0.04$) and higher anxiety ($r = 0.31$; $p = 0.03$) were related to a higher level of frustration in the mother. In a study that examined the effect of the quality of the baby's cry on the mother's interpretation of the baby's challenging temperament, mothers with higher empathy interpreted the baby's temperament as easier and less annoying ($r = 0.25$; $p < 0.05$) than mothers with low empathy. In a study investigating mother's aggression towards her child, a parent's lower cognitive and affective empathy predicted a negative attitude towards the child's behavior and had a moderate relationship with the risk of child maltreatment ($\beta = -0.28$; $p < 0.01$). A higher ability to empathize was connected to greater social support of the child ($r = -0.29$; $p < 0.001$) as well as life control and confidence in one's own abilities ($r = -0.28$; $p < 0.001$). Low empathy was moderately related ($r = 0.3–0.5$) to a weakened ability to restore emotional balance after a distressing situation and to a reduced tolerance for frustration and discomfort. In a study examining the interaction between the characteristics of the mother and the baby, the mother's ability to empathize was connected to the ability to perceive the baby's cues ($r = 0.65$; $p < 0.001$). In the same study, mothers reported higher empathy than fathers ($t = 3.66$; $p < 0.001$) and a more positive attitude towards baby care. In a study investigating babies' reactions to the mother's emotional expressions, the mother's higher empathy had a protective effect on the fearful babies. Empathy reduced babies' sensitivity to fearful stimuli, which helped the baby cope better ($F = 6.52$; $p = 0.019$). In another study, the mother's higher empathy was connected to the baby's more focused attention ($r = 0.30$; $p < 0.01$) and less tendency to get angry ($r = 0.26$; $p < 0.05$).	
Myers W, Lee E, Montplaisir R, et al. 2021. USA, Hong Kong and Canada. [93]	Revenge filicide characteristics of the killers	Systematic review (n = 62 killers)	**The parent's psychological illness appears to be a risk factor for filicide motivated by revenge.**<br><br>56% of the parents who killed a child with the intention of revenge had some kind of mental illness, for example personality disorder (34%) or antisocial personality disorder	**B***<br><br>**8/11****

The studies were conducted in the United States (n = 43), Canada (n = 5), the United Kingdom (n = 5), Australia (n = 4), India (n = 2), Hong Kong (n = 1) Scotland (n = 1) and in the British Overseas Territory (n = 1)	The data of the killers consisted of national register materials of different countries (n = 52) and materials of forensic psychiatric hospitals (n = 10).	Casestudy<br>The characteristics of the subjects were described using prevalence (%).	(21%). 10% had any substance use disorder and 3% had an anxiety disorder.<br>**The parent's relationship problems and a history of family violence appear to be risk factors for filicide motivated by revenge.**<br>53% of the parents who killed their child in revenge, i.e. in a quarrelsome, estranged or divorce-proceeding marriage. 6% lived in cohabitation and also suffered from relationship problems. 12% were divorced and 21% had separated from cohabitation.<br>The motive for the child's death was revenge against the other parent or the grandparent acting as guardian. In 39% of the cases, the killer felt that the reason was rejection or the end of the relationship. In some cases, domestic violence was involved in ending the relationship. In some of the cases, the killer had threatened to kill the child or spouse before the killing. In 32%, the motive for the death was a custody or visitation rights dispute. In 18%, the motive was the spouse's infidelity or success in life after the divorce. In 11%, the motive was a dispute, for example about money, commitment to a relationship or feeling trapped.	
Yates G, Bass C. 2017. UK. [95]<br>Studies were conducted in the United States (n = 302), Europe (n = 434), Asia (n = 50), Australia (n = 6) and New Zealand (n = 3)	Perpetrators of child medical abuse -characteristics and consequences of maltreatment	Systematic review (n = 796 cases/ 250 studies)<br>Casestudy<br>clinical case studies (n = 309) and case series (n = 487).	**Deliberately causing health problems for the child is likely to disturb the child's school attendance, lead to unnecessary medical examinations and may even result in death.**<br>Parents who maltreat their child caused the child's death by their actions (n = 27/354; 7.6%). In addition, they achieved financial gain by making the child sick (n = 26/377; 6.9%), exposed their child to unnecessary health examinations and visits (n = 110/309; 35.6%) and their actions significantly disrupted the child's schooling (n = 38/309; 12.3%).	**B***<br>**7/11****
Chiesa A, Kallechey L, Harlaar N, et al. 2018. USA and UK. [3]<br>The studies were conducted in the USA	Intimate partner violence and parenting<br>The participants belonged to a lower socioeconomic class. In 22 of the studies, children between	Systematic review and meta-analysis (n = 33 studies)<br>case-control studies (n = 4)<br>cross-sectional studies (n = 19)	**Intimate partner violence may be linked to an increased risk of child maltreatment.**<br>Higher violence scores were associated with greater physical aggression (r = 0.17; 95% CI [0.11, 0.23]), neglect (r = 0.12; 95% CI [0.01, 0.23]) and psychological aggression (r = 0.23; 95% CI [-0.94, 0.47]). A connection was also found between being a victim of intimate partner violence and reduced communication, connectedness, inadequate parenting skills	**C***<br>**11/11****

	the ages of 0 and 5 were examined, and in 20 also children over the age of 5. Those investigated were families (n = 7,173), mothers (n = 13,646) or victims of violence (n = 6,158). The researched victims of intimate partner violence were almost always women.	prospective cohort studies and longitudinal studies (n = 10)	and increased authoritarian upbringing. A negative correlation was found between domestic violence and positive parenting (mean r = -0.08; 95% CI [-0.12, -0.04] p < 0.01), whereby higher scores were associated with less positive parenting.<br><br>The narrative synthesis of the systematic review confirmed the results of the meta-analysis. In accordance with the meta-analysis, in all narratively analyzed studies, at least a partial connection was found between being a victim of intimate partner violence and weakened positive parenting. Victimization was related to negative parenting characteristics in 11/12 studies.	
Vu N, Jouriles E, McDonald R, et al. 2016. USA. [101]<br><br>Origin of studies: N/A	Children's exposure to intimate partner violence and associations with child adjustment problems<br><br>There were participants from risk groups and non-risk groups.	Systematic review and meta-analysis (n = 74 studies)<br><br>longitudinal studies	**Exposure to intimate partner violence appears to increase behavioural problems in children.**<br><br>A child's exposure to intimate partner violence predicts later self-regulation and adjustment problems, such as internalising and externalising problems. An association was found between exposure to intimate partner violence and children's adjustment problems: externalising problems (b = 0.006; SE = 0.002; p = 0.004), internalising problems (b = 0.008; SE = 0.002; p < 0.001) and behavior problems as a whole (b = 0.004; SE = 0.007; p = 0.523). The average age of the children at the time of the domestic violence assessment varied between before birth (during the third trimester of pregnancy) and 18 years. When the child was 5 years old, the association between intimate partner violence and children's externalizing behavior problems was greater (r = 0.21) than when the child was 10 years old (r = 0.13) or 15 years old (r = 0.06) . The age at the time of the assessment of the child's behavior varied between nine months and 27 years. Age had a positive relationship with the correlation of intimate partner violence and internalising problems (QB (1) = 8.65; p = 0.003). When the child was 15 years old, the association between intimate partner violence and children's internalising problems was greater (r = 0.11) than when the child was 10 years old (r = 0.08) or 5 years old (r = 0.06) . Relationship violence, externalising problems (QB (1) = 1.48; p = 0.224), internalising problems (QB (1) = 10.79; p = 0.373), both behavior problems (QB (1) = 0.77; p = 0.382).	**B***<br><br>**10/11****
Fong V, Hawes D, Allen J. 2019. UK and Australia. [86]	Risk and protective factors of externalizing problems in children	Systematic review (n = 31 studies)	**Exposure to intimate partner violence appears to increase behavioural problems in children.**	**B***<br><br>**9/11****

Origin of studies: N/A	exposed to intimate partner violence<br><br>the study analysed narratively 1) the relationship of domestic violence perpetrated by fathers to the child's age, gender, expression of emotions and assessment of the experience of violence, and 2) the relationship between the mental health and maternal warmth of a mother who experienced intimate partner violence and the child's externalising behavioural problems. In addition, protective factors were examined.	meta-analyses (n = 2)<br>cross-sectional studies (n = 13)<br>cross-sectional studies/RCT studies (n = 1)<br>cross-sectional studies (clinical trial) (n = 1)<br>longitudinal studies (n = 12)<br>longitudinal studies/cohorts (n = 1)<br>prospective studies (n = 1).	The importance of the child's age when he was exposed to intimate partner violence varied between different studies. Younger age of the child at the time of first exposure was significantly associated with increased externalising behavioural problems, but severity of exposure to violence had the greatest effect. The importance of the child's gender varied between different studies. There was no difference between boys and girls in the occurrence of externalizing behavior problems.<br><br>**Protective factors appear to prevent behavioural problems in children.**	
Timshel I, Montgomery E, Dalgaard N. 2017. Denmark. [85]<br><br>The studies were conducted in the United States (n = 8), Australia (n = 2), the Netherlands (n = 2), Canada (n = 1), Sweden (n = 1) and Vietnam (n = 1)	Risk and protective factors associated with family-related violence in refugee families<br><br>The refugee families' country of origin was Asia, Africa, the Middle East, South and Central America and Europe.	Systematic review (n =15 studies)<br><br>longitudinal studies (n = 1)<br>cross-sectional studies (n = 1)<br>comparative cohort studies (n = 1)<br>quantitative report-based studies (n = 2)<br>qualitative studies (n = 4)<br>mixed-method studies (n = 6).	**Asylum seekers' families may have risk factors for family violence. C**<br><br>Risk factors for domestic violence were trauma experienced by parents, abuse experienced by parents in childhood, trauma experiences and resulting mental health problems, such as depression and PTSD (Post Traumatic Stress Disorder), stress, unemployment, substance use (alcohol), low level of education, language barrier, inequality feelings, problems of interaction between parents and children, parenting problems, single parenthood, relationship problems, divorce, large family size, problems of adapting to the family's culture, relationship conflicts caused by adapting to the culture (patriarchal beliefs, values and norms, use of violence and punishment acceptance in one's own culture, the change in power relations between women and men in the new culture), weak socio-economic status of the family, lack of knowledge and education.<br><br>**Protective factors appear to prevent behavioural problems in children. B**<br><br>The protective factors were parents' positive coping strategies, parents' education, time spent in the country of arrival and supportive neighborly relations.	**C*/B***<br><br>**9/11****
Botha E, Joronen K, Kaunonen M. 2019. Finland. [9]	The consequences of having an excessively crying infant in the family	Integrative review (n =31 studies)	**Incessant or excessive crying by the baby appears to potentially cause negative effects on the family. (B)**	**B***<br><br>**10/11****

The studies were conducted in the United States (n = 9), Sweden (n = 3), Turkey (n = 3), Japan (n = 2), Denmark (n = 1), the United Kingdom (n = 1), Brazil (n = 1), the Netherlands (n = 1), Estonia (n = 1), Ireland (n = 1), and Germany (n = 1)	Participants were parents	cohort studies with a longitudinal design (n = 11)<br>RCT studies (n = 4)<br>cross-sectional studies (n = 5)<br>case-control studies (n = 1)<br>case-control studies (n = 1)<br>descriptive quantitative (n = 1)<br>and qualitative studies (n = 8)	Ten themes were formed in the integrative review. The baby's very profuse crying caused despair in the family, ruined everyday life, hindered breastfeeding, isolated parents to loneliness, strained and broke family relationships, caused feelings of parenting failure, led to physical and psychological exhaustion, could put the baby at risk of maltreatment or even infanticide and increase problems later in the child's life.	
Barreto T, Bento M, Jagersbacher J, et al. 2020. Brazil. [100]<br><br>The studies were conducted in Australia (n = 2), Bosnia and Herzegovina (n = 1), Brazil (n = 3), Spain (n = 1), the Netherlands (n = 1), Turkey (n = 2), Iran (n = 1), Jordan (n = 1), Great Britain (n = 1) and Serbia (n = 1)	Prevalence of depression, anxiety and substance-related disorders in parents of children with cerebral palsy<br><br>The number of participants varied between studies. A total of 1,264 mothers and 105 fathers.	Systematic review (n = 14 studies)<br><br>cross-sectional studies. Five studies also included a control group.	**The emotional and behavioural problems of children with cerebral palsy (CP) appear to increase stress and depression in parents, and the parents also appear to receive little social support. (B)**<br><br>There was a correlation (r = 0.329) between the scores obtained on the Perceived Stress Scale (PSS) of the parent of a child with CP, depression and the experience of little support from the family, and the scores on the Strengths and Difficulties Questionnaire (SDQ) measuring the child's mental well-being and behavior ; p < 0.005; r = 0.246; p < 0.01; r = 0.185; p = 0.04). Those parents whose child had emotional and behavioral problems were more likely to experience increased stress and depression (psychological distress) and felt that they received less support from their family. In addition, the results showed that there was a significant negative relationship between parental stress, depression and social support assessed with the Multidimensional Scale of Perceived Social Support (MSPSS) (r = -0.389; p < 0.0005; r = -0.379; p < 0.0005). This shows that parents who experienced high stress and depression felt that they received little social support.	**B***<br><br>**10/11****
Clayton K, Lee JB, Cheung K, et al. 2018. Canada. [102]<br><br>Studies were conducted in the United States (n = 14), Canada (n = 2), China (n = 1) and Palestine (n = 1)	Quantifying the relationship between Attention-Deficit/Hyperactivity disorder and experiences of child maltreatment<br><br>Participants were between 7 and 42 years old at the time of the study. The number of participants was not reported.	Systematic review and meta-analysis (n = 18 studies)<br><br>cohort studies	**Children with ADHD appear to experience more maltreatment than other children. (B)**<br><br>A connection was found between ADHD (Attention Deficit Hyperactivity Disorder) and experiences of maltreatment (r = 0.28; p < 0.001). Participants with ADHD had experienced maltreatment more often as children compared to those without ADHD (OR = 2.39; p < 0.001). Of those who experienced maltreatment (n = 5 studies), 45.9% had ADHD. Comparing different forms of abuse, 26.2% had experienced physical abuse (n = 9 studies), sexual violence (n = 8 studies) 14%, neglect (n = 4 studies) 24.4% and emotional abuse (n = 4 research) 57.7%. ADHD was also associated with a greater number of maltreatment experiences (d = 0.40; p < 0.001). Those with ADHD had experienced maltreatment 2.39 times	**B***<br><br>**11/11****

			more often than subjects in the control group (p < 0.001). They had experienced physical abuse 2.02 times more (p = 0.005), neglect 5.33 times more (p < 0.001) and emotional abuse 8.59 times more (p < 0.001). No significant differences emerged in the group comparisons regarding the forms of maltreatment regarding the witnessing of sexual violence and intimate partner violence. Those who had symptoms of ODD (Oppositional Defiant Disorder) or CD (Conduct Disorder) in addition to ADHD had experienced maltreatment more often than those who only had ADHD (OR = 2.55; p = 0.004).	
Craig S, Bondi B, O'Donnell K, et al. 2020. Canada and USA. [103]<br><br>Studies were conducted in the United States (n = 14), South America (n = 4), Asia (n = 7), Africa (n = 1) and Europe (n = 9)	ADHD and exposure to maltreatment in children and youth<br><br>Participants were between 0 and 20 years old at the time of the study (average age < 18). The number of subjects varied between different studies (n = 11–8192).	Systematic review (n = 35 studies)<br><br>prevalence and longitudinal studies	**Children with ADHD appear to experience more maltreatment than other children. (B)**<br><br>A connection was found between ADHD and maltreatment. ADHD was diagnosed in 9 % of the children who were clients of child protection who experienced maltreatment, in 18.6 % of the children suspected of being maltreated, and in 50 % of the adopted children who experienced severe maltreatment in their birth home. The children had experienced physical or emotional neglect in their birth home, 49 % physical abuse and 20 % sexual violence. 55 % of the children abused in the foster home had been diagnosed with ADHD. 20 % of them had ADHD without ODD) and 35 % had ODD in addition to ADHD. 74–85.7 % of children with FASD (Fetal Alcohol Syndrome Disorder) were also diagnosed with ADHD. 86.73% of FASD children had experienced neglect, 50% physical, 23.47% verbal and 46.94 % sexual abuse compared to children not diagnosed with FASD. 10 studies report the prevalence and risks of childhood maltreatment experiences in ADHD children. 76.7 % of ADHD children had experienced neglect, 53.5 % emotional and 46.7 % physical abuse compared to children who had not experienced abuse. In one study, children who experienced PTSD (Post Traumatic Stress Disorder) were found to have a 3.5 times risk of developing ADHD compared to those who did not experience PTSD. In another study, children who had experienced emotional abuse had 11 times the risk of developing ADHD compared to children who had not experienced emotional abuse. in the third study, children exposed to maltreatment had 3.5 times the risk of developing ADHD compared to children who had not been exposed to maltreatment. Multiple adverse childhood experiences before age 9 predicted an increased risk of ADHD at age 9. The results broadly suggest that children with ADHD experience maltreatment more often than	**B***<br><br>**8/11****

			children without ADHD. Studies examining genetics did not find significant connections between ADHD and maltreatment. Results were variable between longitudinal studies (n = 5). Some studies showed that maltreatment increases ADHD symptoms later in life. For example, experiencing multiple forms of maltreatment between ages 0 and 12 predicted ADHD symptoms at ages 12 and 14. Child-reported PTSD symptoms at age 12 were indirectly related to different forms of maltreatment experienced between ages 0 and 12, such as exposure to physical abuse and interparental violence, and the presence of ADHD symptoms at age 14.<br><br>Longitudinal studies provide preliminary support for the hypothesis that early childhood maltreatment increases the risk of ADHD symptoms and diagnosis during childhood and adolescence. In studies examining parental behavioral characteristics (n = 7), in one study, hyperactivity and impulsivity in mothers of ADHD children increased the risk of child emotional abuse and neglect. The mother's history of physical neglect and the father's attention deficit disorder increased the child's risk of sexual violence, but the mother's emotional maltreatment decreased it. The mother's positive attitude towards verbal discipline and physical discipline and their use were related to increased children's ADHD symptoms and the child's aggressive behavior. Studies examining child aggressive behavior (n = 4) found that emotional and physical maltreatment were associated with increased aggressive behavior in boys with ADHD. Exposure to severe maltreatment and trauma predicted arrests later in life. 54.5 % of young people with trauma experiences were arrested compared to young people without trauma experiences (22.4 %).	

*Level of evidence according to JBI criteria: (A) Strong research evidence, (B) Moderate research evidence, (C) Little research evidence, (D) No research evidence available.

** The JBI Critical Appraisal Tool for Use in JBI Systematic Reviews is available at: https://jbi.global/sites/default/files/2019-05/JBI_Critical_Appraisal-Checklist_for_Systematic_Reviews2017_0.pdf
